# Identification of the promising oleaster (*Elaeagnus angustifolia* L.) genotypes based on fruit quality‐related characters

**DOI:** 10.1002/fsn3.2536

**Published:** 2021-08-16

**Authors:** Leila Safdari, Ali Khadivi

**Affiliations:** ^1^ Department of Horticultural Sciences Faculty of Agriculture and Natural Resources Arak University Arak Iran

**Keywords:** breeding, *Elaeagnus angustifolia* L., fruit weight, gene pool, superior accessions

## Abstract

The fruits of oleaster (*Elaeagnus angustifolia* L.) are rich in nutritional value and contain protein, sugar, vitamins, and minerals. The present investigation was performed to evaluate the morphological variability of the naturally grown accessions of this species. There was considerable variation among the accessions based on all the traits measured. The range of fruit weight was from 0.32 to 3.04 g, with an average of 1.48. Fruit yield was significantly and positively correlated with tree growth vigor, canopy density, branching, branch density, and leaf density. Principal component analysis (PCA) indicated nine components of data accounted for 74.93% of the total variance. Ward cluster analysis using Euclidean distance classified the accessions into two main clusters and showed significant differences among the accessions studied. Among the area studied, 14 accessions showed the highest value of fruit quality‐related characters, which are suitable for fresh consumption and health benefits. The results provided important information useful for selecting the preferred accessions for commercial cultivation and breeding programs.

## Graphical abstract

Ward cluster analysis using Euclidean distance classified the accessions into two main clusters and showed significant differences among the accessions studied in the characters measured. Among the area studied, 14 accessions showed the highest fruit weight value suitable for fresh consumption and health benefits.

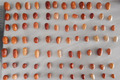

## 1 INTRODUCTION

Oleaster (*Elaeagnus angustifolia* L.) belongs to the family Elaeagnaceae and is a deciduous tree or shrub resembling an olive tree with small and reddish brown fruits (Fonia et al., 2009; Sahan et al., 2013). This plant's life is long and reaches 80–200 years, its growth is rapid that its height reaches 10 meters and 30 cm in diameter, and its fruiting begins after 5–6 years (Kiseleva & Chindyaeva, 2011). This plant has high tolerance to a wide range of adverse environmental conditions such as severe drought, flooding, and rocky and saline soils (Asadiar et al., 2013). Different parts of this tree have many medicinal uses and are used in the perfume industry. Also, its wood is used in woodworking and musical instruments (Kiseleva & Chindyaeva, 2011).

The nutritional value of *E. angustifolia* fruits is considerable, owing to significant amounts of protein, sugar, vitamins, and minerals (Fonia et al., 2009; Taheri et al., 2010). Its fruits are eaten fresh or dried and are rich in vitamin C, thiamin B1, and minerals such as calcium, manganese, iron, potassium, and magnesium. It contains tocopherol and carotene (Boudraa et al., 2010). Phytochemical studies have shown that its fruit extract contains flavonoids, polysaccharides, cardiac, terpenoids, coumarins, phenol carboxylic acids, amino acids, saponins, carotenoids, vitamins, and tannins (Ayaz & Bertoft, 2001; Natanzi et al., 2012; Okmen & Turkcan, 2013). Its flowers are small, aromatic, and yellowish white and are used as a source of nectar for honeybees and as a flavoring agent in liqueur producers (Fonia et al., 2009; Kiseleva & Chindyaeva, 2011). Furthermore, in traditional medicine, its flowers are used to treat tetanus (Wang et al., 2013).

Genetic variation generated and maintained by evolution is an inherited diversity among populations (Demol et al., 2001) and is also an important intrinsic trait that helps breeders develop breeding programs. Phenotypic description of plants has been introduced as an efficient method for identifying elite individuals. This method is an important tool for selecting varieties or lines based on morphological and agronomic traits (Bajracharya et al., 2006; Ndour, 1998).

A proper characterization of genetic resources is necessary to develop breeding strategies. Native genetic resources are well‐adapted and also are interesting gene pools for breeding programs. The first step in the description and classification of the plants is morphological characterizations. Statistical methods such as principal component and cluster analyses are useful tools for describing and screening accessions in a germplasm collection (Peeters & Martinelli, 1989).

The study of food‐drug fruit species can help protect them as well as their use in breeding programs and health‐related industries. The present study was performed to evaluate the phenotypic diversity of naturally grown accessions of *E. angustifolia* to select superior accessions in terms of fruit quality.

## 2 MATERIALS AND METHODS

### 2.1 Plant material

The present study was performed to evaluate the phenotypic diversity of 84 naturally grown accessions of *E. angustifolia* to select superior accessions in terms of fruit quality. The plant materials studied were collected from four areas of Markazi province in Iran. Astaneh area is located at 34˚50'33"N latitude, 49˚22'22"E longitude, and 2010 m height above sea level. Dehsad area is located at 34˚53'39"N latitude, 49˚43'51"E longitude, and 1920 m height above sea level. Senejan area is located at 34˚51'23"N latitude, 49˚23'19"E longitude, and 1836 m height above sea level. Dehmahdi area is located at 34˚49'37"N latitude, 49˚27'44"E longitude, and 2097 m height above sea level. The accessions examined were named according to their location, and the names were supplemented with numerical characters. To reduce the error, preventing the collection of the clones belonging to an accession and ultimately increasing accuracy, a proper distance of 200 m between trees in each area was considered.

### 2.2 The characters evaluated

In total, 28 morphological characters (Table 1) were used for phenotypic diversity of the accessions studied. Fifty replicates for leaf and fruit were used for measurements, and the mean values were used for analysis. Leaf length, leaf width, petiole length, petiole thickness, thorn length on annual shoot, thorn base thickness on annual shoot, fruit length, fruit width, fruit stalk length, fruit stalk diameter, fruit flesh thickness, stone length, and stone width were measured by a digital caliper. Fruit fresh weight, fruit dry weight, and stone weight were measured by using an electronic balance with 0.01 g precision. In addition, the rest traits (Table 2) were qualitatively estimated based on rating and coding.

**TABLE 1:** Descriptive statistics for morphological traits utilized in the studied accessions of *E. angustifolia*

No.	Character	Abbreviation	Unit	Minimum	Maximum	Mean	*SD*	CV (%)
1	Tree growth habit	TGH	Code	1	9	3.43	2.45	71.31
2	Tree growth vigor	TGV	Code	1	5	3.71	1.19	32.02
3	Tree height	TH	Code	1	5	4.14	1.21	29.32
4	Trunk color	TrC	Code	1	7	2.62	2.16	82.52
5	Trunk diameter	TrD	Code	1	5	3.67	1.71	46.59
6	Canopy density	CaDe	Code	1	5	2.88	1.56	54.27
7	Branching	Br	Code	1	5	4.10	1.22	29.73
8	Branch density	BrDe	Code	1	5	3.98	1.34	33.54
9	Branch flexibility	BrF	Code	1	5	3.83	1.68	43.81
10	Thorn presence	ThP	Code	0	1	0.33	0.47	143.64
11	Leaf density	LDe	Code	1	5	2.88	1.59	55.35
12	Leaf shape	LSh	Code	1	7	4.17	2.56	61.49
13	Leaf upper surface color	LUSC	Code	1	5	3.12	1.65	52.98
14	Leaf lower surface color	LLoSC	Code	1	5	4.62	1.14	24.68
15	Leaf apex shape	LASh	Code	1	3	1.88	1.00	53.14
16	Leaf length	LLe	mm	39.81	105.96	64.10	11.59	18.08
17	Leaf width	LWi	mm	17.25	44.60	26.48	5.68	21.46
18	Petiole length	PeLe	mm	4.44	14.32	9.22	2.30	24.98
19	Fruit yield	FrYi	Code	1	5	3.93	1.50	38.24
20	Fruit length	FrLe	mm	12.67	31.04	23.59	4.46	18.90
21	Fruit width	FrWi	mm	8.24	20.22	14.95	2.13	14.23
22	Fruit weight	FrWe	g	0.32	3.04	1.48	0.55	37.39
23	Fruit shape	FrSh	Code	1	5	3.55	1.62	45.75
24	Fruit color	FrC	Code	1	7	5.05	2.24	44.32
25	Fruit taste	FrTa	Code	1	7	4.19	1.92	45.75
26	Seed length	SeLe	mm	12.11	29.12	20.74	4.01	19.35
27	Seed width	SeWi	mm	4.57	8.77	5.67	0.74	13.04
28	Seed weight	SeWe	g	0.17	0.75	0.43	0.13	30.56

**TABLE 2:** Frequency distribution for the measured qualitative morphological characters in the studied accessions of *E. angustifolia*

Character	Frequency (no. of accessions)					
	0	1	3	5	7	9
Tree growth habit	‐	Weeping (33)	Spreading (19)	Open (16)	Semi‐erect (13)	Erect (3)
Tree growth vigor	‐	Low (5)	Intermediate (44)	High (35)	‐	‐
Tree height	‐	Low (5)	Intermediate (26)	High (53)	‐	‐
Trunk color	‐	Gray (51)	Cupric (5)	Red (21)	Black (7)	‐
Trunk diameter	‐	Low (21)	Intermediate (14)	High (49)	‐	‐
Canopy density	‐	Low (28)	Intermediate (33)	High (23)	‐	‐
Branching	‐	Low (5)	Intermediate (28)	High (51)	‐	‐
Branch density	‐	Low (8)	Intermediate (27)	High (49)	‐	‐
Branch flexibility	‐	Low (19)	Intermediate (11)	High (54)	‐	‐
Thorn presence	Absent (56)	Present (28)	‐	‐	‐	‐
Leaf density	‐	Low (29)	Intermediate (31)	High (24)	‐	‐
Leaf shape	‐	Wide ovate (27)	Oblong (12)	Narrow lanceolate (14)	Lanceolate (31)	‐
Leaf upper surface color	‐	Light green (26)	Green (27)	Dark green (31)	‐	‐
Leaf lower surface color	‐	Light green (7)	Silver‐green (2)	Silver (75)	‐	‐
Leaf apex shape	‐	Blate (47)	Acute (37)		‐	‐
Fruit yield	‐	Low (13)	Intermediate (19)	High (52)	‐	‐
Fruit shape	‐	Round (19)	Oval (23)	Oblong (42)	‐	‐
Fruit color	‐	Cream (7)	Yellow (30)	Orange (1)	Brown (46)	‐
Fruit taste	‐	Astringent (11)	Slightly sweet (29)	Sweet (27)	Very sweet (17)	‐

### 2.3 Statistical analysis

The meaningful differences between the accessions in terms of the traits measured were determined using one‐way analysis of variance (ANOVA) by SAS software (SAS Institute, 1990). The parameters, including minimum, maximum, mean, standard deviation, and coefficient of variation (CV), were calculated. The Pearson correlation coefficient was used to determine the correlation between traits with SPSS^®^ software version 16 (SPSS Inc. Norusis, 1998). The relationship between the accessions was analyzed with principal component analysis (PCA) using SPSS software. Cluster analysis was performed using the Euclidean distance coefficient and Ward method with PAST software (Hammer et al., 2001). Distance coefficients were standardized using Z scale. In addition, the first and second principal components (PC1 and PC2) were used to generate the scatterplot with PAST software.

## 3 RESULTS AND DISCUSSION

There was considerable variation among the accessions based on all the traits measured. The highest CVs belonged to thorn presence (143.64%), trunk color (82.52%), tree growth habit (71.31%), leaf shape (61.49%), leaf density (55.35%), canopy density (54.27%), leaf apex shape (53.14%), and leaf upper surface color (52.98%). In contrast, seed width (13.04%), fruit width (14.23%), leaf length (18.08%), fruit length (18.90%), and seed length (19.35%) showed the lowest CVs (Table 1). The traits with high CV increase the chance of selection among the accessions, and the traits with low CV show homology and stability among the accessions.

Five types of tree growth habits were observed, including weeping (33 accessions), spreading (19), open (16), semi‐erect (13), and erect (3) (Table 2). Tree growth vigor, canopy density, and leaf density were predominantly intermediate, and tree height, trunk diameter, branching, branch density, branch flexibility showed high value. Thorn was observed in 28 out of 84 accessions. The accessions were clustered into four groups based on leaf shape, including wide ovate (27 accessions), oblong (12), narrow lanceolate (14), and lanceolate (31). Leaf length ranged from 39.81 to 105.96 mm, leaf width varied from 17.25 to 44.60 mm, and petiole length range between 4.44 and 14.32 mm. Khadivi (2018) reported the range of 32.00–86.00 mm for leaf length, 7.20–23.43 mm for leaf width, and 4.50–15.73 mm for petiole length in *E. angustifolia* from an area of Markazi province in Iran, whereas Khadivi et al. (2020) reported the range of 32.06–82.07 mm for leaf length, 8.72–26.58 mm for leaf width, and 5.26–16.48 mm for petiole length in this species from six areas of Isfahan province in Iran.

Fruit yield was predominantly high (52 accessions). Three types of fruit shapes were observed, including round (19), oval (23), and oblong (42). Fruit length ranged from 12.67 to 31.04 mm, and the range of fruit width was from 8.24 to 20.22 mm, and fruit weight ranged between 0.32 and 3.04 g with a mean of 1.48 (Table 1). Khadivi (2018) reported the range of 19.69–31.90 mm for fruit length, 11.60–19.72 mm for fruit width, and 0.93–5.53 g for fruit weight in *E. angustifolia* from an area of Markazi province in Iran, whereas Khadivi et al. (2020) reported the range of 12.30–28.89 mm for fruit length, 9.82–20.05 mm for fruit width, and 0.46–3.25 g for fruit weight in this species from six areas of Isfahan province in Iran.

**FIGURE 1:**
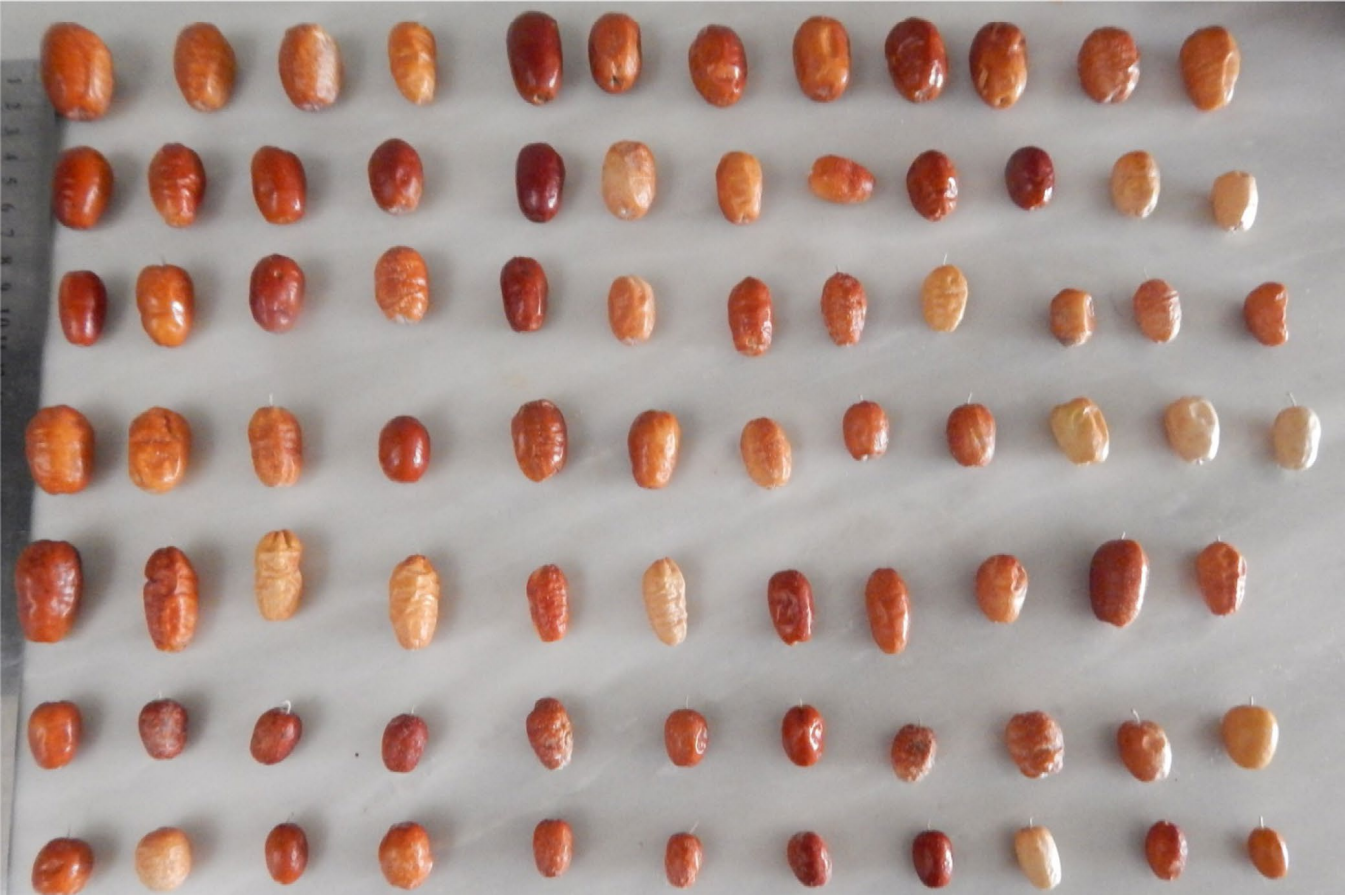
Fruits of *E. angustifolia* accessions studied

Brown fruit color was predominant (46) and followed by yellow (30). Fruit taste was astringent in 11, slightly sweet in 29, sweet in 27, and very sweet in 17 accessions. Khadivi (2018) reported that fruits in the majority of *E. angustifolia* genotypes had a sweet taste. Seed length ranged from 12.11 to 29.12 mm, seed width varied from 4.57 to 8.77 mm, and the range of seed weight was 0.17–0.75 g. The fruit's pictures of the studied accessions of *E. angustifolia* are shown in Figure 1.

There were significant correlations between some characters (Table 3). Tree growth vigor was positively and significantly correlated with tree height (*r* = 0.36), trunk diameter (*r* = 0.64), canopy density (*r* = 0.53), branching (*r* = 0.48), branch density (*r* = 0.46), and leaf density (*r* = 0.50), in accordance with the previous findings in *E. angustifolia* (Khadivi, 2018; Khadivi et al., 2020). Fruit yield was significantly and positively correlated with tree growth vigor (*r* = 0.29), canopy density (*r* = 0.27), branching (*r* = 0.28), branch density (*r* = 0.33), and leaf density (*r* = 0.22) and corresponded with the previous results in *E. angustifolia* (Khadivi, 2018; Khadivi et al., 2020). Fruit weight had positive and significant correlations with fruit length (*r* = 0.78), fruit width (*r* = 0.92), fruit shape (*r* = 0.54), fruit taste (*r* = 0.43), seed length (*r* = 0.71), seed width (*r* = 0.45), and seed weight (*r* = 0.88) and agreed with the previous results in *E. angustifolia* (Khadivi, 2018; Khadivi et al., 2020).

**TABLE 3:** Simple correlations among the morphological variables utilized in the studied accessions of *E. angustifolia*

Character	TGH	TGV	TH	TrC	TrD	CaDe	Br	BrDe	BrF	ThP	LDe	LSh	LUSC	LLoSC	LASh	LLe	LWi	PeLe	FrYi	FrLe	FrWi	FrWe	FrSh	FrC	FrTa	SeLe	SeWi	SeWe
TGH	1																											
TGV	−0.09	1																										
TH	0.17	0.36**	1																									
TrC	−0.06	0.13	−0.14	1																								
TrD	−0.05	0.64**	0.55**	0.00	1																							
CaDe	−0.23*	0.53**	0.37**	0.07	0.33**	1																						
Br	−0.33**	0.48**	0.12	−0.04	0.29**	0.42**	1																					
BrDe	−0.27*	0.46**	0.31**	−0.04	0.42**	0.51**	0.69**	1																				
BrF	−0.07	−0.04	−0.02	0.14	−0.05	0.11	0.14	0.11	1																			
ThP	0.08	−0.13	−0.08	0.15	−0.33**	−0.04	−0.10	−0.03	0.10	1																		
LDe	−0.17	0.50**	0.39**	0.09	0.40**	0.92**	0.36**	0.46**	0.13	−0.07	1																	
LSh	−0.25*	0.06	0.02	0.05	0.08	−0.03	0.05	0.13	−0.15	−0.11	−0.05	1																
LUSC	−0.05	−0.17	0.10	−0.06	−0.01	−0.16	−0.09	−0.01	0.19	−0.14	−0.12	−0.01	1															
LLoSC	−0.03	0.10	−0.10	0.06	−0.07	0.14	−0.04	−0.07	0.04	0.02	0.16	−0.01	−0.13	1														
LASh	−0.12	0.03	−0.05	0.07	0.08	−0.06	−0.01	0.03	−0.16	−0.02	−0.05	0.66**	−0.09	0.09	1													
LLe	−0.07	0.13	0.16	−0.06	0.17	0.04	−0.01	0.13	0.14	−0.13	0.01	0.03	0.28**	−0.08	0.14	1												
LWi	0.15	0.12	−0.03	0.07	0.02	−0.07	−0.13	−0.06	0.14	−0.03	−0.06	−0.51**	0.12	−0.07	−0.46**	0.37**	1											
PeLe	−0.06	0.10	0.10	−0.03	0.11	0.12	0.04	0.14	0.19	−0.16	0.10	0.18	0.01	−0.15	0.22*	0.53**	0.00	1										
FrYi	−0.01	0.29**	0.10	0.08	0.08	0.27*	0.28**	0.33**	−0.02	0.10	0.22*	0.04	−0.22*	−0.07	−0.17	−0.03	0.04	0.09	1									
FrLe	−0.11	0.07	−0.21*	0.00	−0.05	−0.08	0.17	0.08	−0.14	−0.04	−0.10	0.00	0.11	−0.08	−0.08	0.11	0.10	−0.09	0.33**	1								
FrWi	−0.07	0.09	−0.10	−0.04	−0.05	−0.02	0.16	0.07	−0.18	−0.11	−0.02	−0.05	0.08	0.08	−0.07	0.09	0.06	0.01	0.25*	0.76**	1							
FrWe	−0.08	0.14	−0.11	0.04	0.00	0.05	0.16	0.12	−0.18	−0.17	0.05	−0.02	0.06	0.06	−0.05	0.06	0.05	0.04	0.34**	0.78**	0.92**	1						
FrSh	−0.07	0.02	−0.24*	−0.01	−0.08	−0.01	0.21	0.06	0.01	−0.02	−0.01	0.04	0.01	0.01	−0.03	0.07	0.06	−0.03	0.30**	0.75**	0.46**	0.54**	1					
FrC	0.08	0.08	0.00	−0.01	−0.13	−0.01	−0.14	−0.16	−0.08	0.19	−0.05	−0.15	−0.31**	−0.03	−0.13	−0.25*	−0.12	0.00	0.16	−0.01	0.15	0.11	−0.21*	1				
FrTa	0.16	0.17	−0.07	0.05	0.12	−0.07	0.10	0.09	−0.18	−0.18	−0.06	0.18	0.06	0.01	0.08	−0.11	0.05	−0.05	0.15	0.36**	0.38**	0.43**	0.31**	−0.13	1			
SeLe	−0.10	0.06	−0.23*	−0.01	−0.04	−0.09	0.16	0.08	−0.14	−0.06	−0.11	−0.01	0.12	−0.10	−0.09	0.11	0.12	−0.12	0.33**	0.98**	0.67**	0.71**	0.76**	−0.03	0.36**	1		
SeWi	−0.09	0.11	−0.04	−0.08	0.13	−0.06	0.04	0.00	−0.12	−0.26*	−0.05	0.11	0.03	0.07	−0.01	−0.11	−0.17	−0.03	0.03	0.19	0.52**	0.45**	0.07	0.14	0.29**	0.14	1	
SeWe	−0.05	0.11	−0.17	0.04	−0.04	−0.04	0.14	0.06	−0.22*	−0.09	−0.07	−0.02	0.04	0.04	−0.09	0.02	0.08	−0.11	0.30**	0.86**	0.85**	0.88**	0.63**	0.09	0.45**	0.82**	0.44**	1

Note

For the explanation of morphological character symbols, see Table 1. *, **. Correlation is significant at *p* ≤.05 and .01 levels, respectively.

The PCA method divided the traits into nine independent components, each of which had an eigenvalue higher than 1, justifying 74.93% of the total variance (Table 4). Six traits, including fruit length, fruit width, fruit weight, fruit shape, seed length, and seed weight, were placed in the PC1, which accounted for 19.18% of the total variance. The PC2 accounted for 14.15% of the variance and included tree growth vigor, tree height, trunk diameter, canopy density, branching, branch density, and leaf density. The attributes, including leaf shape, leaf apex shape, and leaf width, were placed in PC3 and accounted for 8.23% of the total variance.

**TABLE 4:** Eigenvalues of the principal component axes from the PCA of morphological characters in the studied accessions of *E. angustifolia*

Character	Component								
	1	2	3	4	5	6	7	8	9
Tree growth habit	−0.08	−0.14	−0.22	0.08	−0.02	−0.09	−0.73**	−0.05	0.02
Tree growth vigor	0.08	0.79**	0.01	0.08	0.08	0.13	−0.14	0.02	0.23
Tree height	−0.25	0.60**	−0.07	−0.01	0.15	0.12	−0.32	−0.13	−0.27
Trunk color	−0.01	0.02	0.02	0.06	−0.01	−0.07	0.05	0.08	0.87**
Trunk diameter	−0.10	0.71**	0.07	−0.20	0.09	0.31	−0.25	−0.14	0.04
Canopy density	−0.03	0.80**	−0.05	0.12	0.05	−0.11	0.22	0.32	−0.04
Branching	0.20	0.63**	0.07	−0.08	−0.16	−0.02	0.45	−0.21	−0.01
Branch density	0.11	0.74**	0.10	−0.09	0.01	−0.05	0.31	−0.24	−0.03
Branch flexibility	−0.20	0.02	−0.32	−0.12	0.24	−0.09	0.50**	−0.01	0.31
Thorn presence	−0.04	−0.16	−0.06	0.34	−0.12	−0.57**	0.06	−0.03	0.15
Leaf density	−0.06	0.79**	−0.07	0.07	0.04	−0.07	0.16	0.36	−0.03
Leaf shape	0.01	0.04	0.87**	−0.09	0.08	0.06	0.06	−0.09	0.08
Leaf upper surface color	0.03	−0.16	−0.19	−0.58**	0.18	0.25	0.16	−0.17	−0.07
Leaf lower surface color	0.01	0.03	0.04	−0.01	−0.11	0.04	0.01	0.86**	0.08
Leaf apex shape	−0.06	−0.04	0.86**	−0.07	0.21	−0.02	−0.05	0.12	0.08
Leaf length	0.09	0.07	−0.06	−0.30	0.85**	−0.04	−0.02	−0.02	−0.05
Leaf width	0.11	−0.01	−0.70**	−0.23	0.28	−0.09	−0.23	−0.03	0.22
Petiole length	−0.06	0.10	0.19	0.12	0.83**	0.05	0.10	−0.11	0.02
Fruit yield	0.42	0.39	−0.03	0.37	0.02	−0.25	−0.01	−0.23	0.11
Fruit length	0.96**	−0.03	−0.03	−0.06	0.01	−0.03	0.03	−0.08	−0.03
Fruit width	0.83**	−0.01	−0.07	0.17	0.08	0.38	0.04	0.09	−0.05
Fruit weight	0.87**	0.07	−0.03	0.15	0.08	0.32	0.01	0.08	0.02
Fruit shape	0.80**	0.00	0.04	−0.21	−0.03	−0.25	0.07	0.03	0.01
Fruit color	−0.02	−0.09	−0.12	0.85**	−0.02	0.15	−0.04	−0.07	−0.03
Fruit taste	0.46	0.10	0.17	−0.20	−0.20	0.29	−0.33	−0.10	0.31
Seed length	0.93**	−0.03	−0.04	−0.11	−0.02	−0.07	0.01	−0.11	−0.02
Seed width	0.27	−0.01	0.08	0.19	−0.10	0.80**	0.08	0.03	0.01
Seed weight	0.92**	−0.01	−0.04	0.08	−0.05	0.23	−0.04	0.04	0.03
Total	5.37	3.96	2.30	1.78	1.78	1.75	1.57	1.27	1.20
% of Variance	19.18	14.15	8.23	6.35	6.35	6.25	5.61	4.54	4.28
Cumulative %	19.18	33.34	41.56	47.91	54.25	60.50	66.11	70.65	74.93

Note

** Eigenvalues are significant ≥0.50.

**FIGURE 2:**
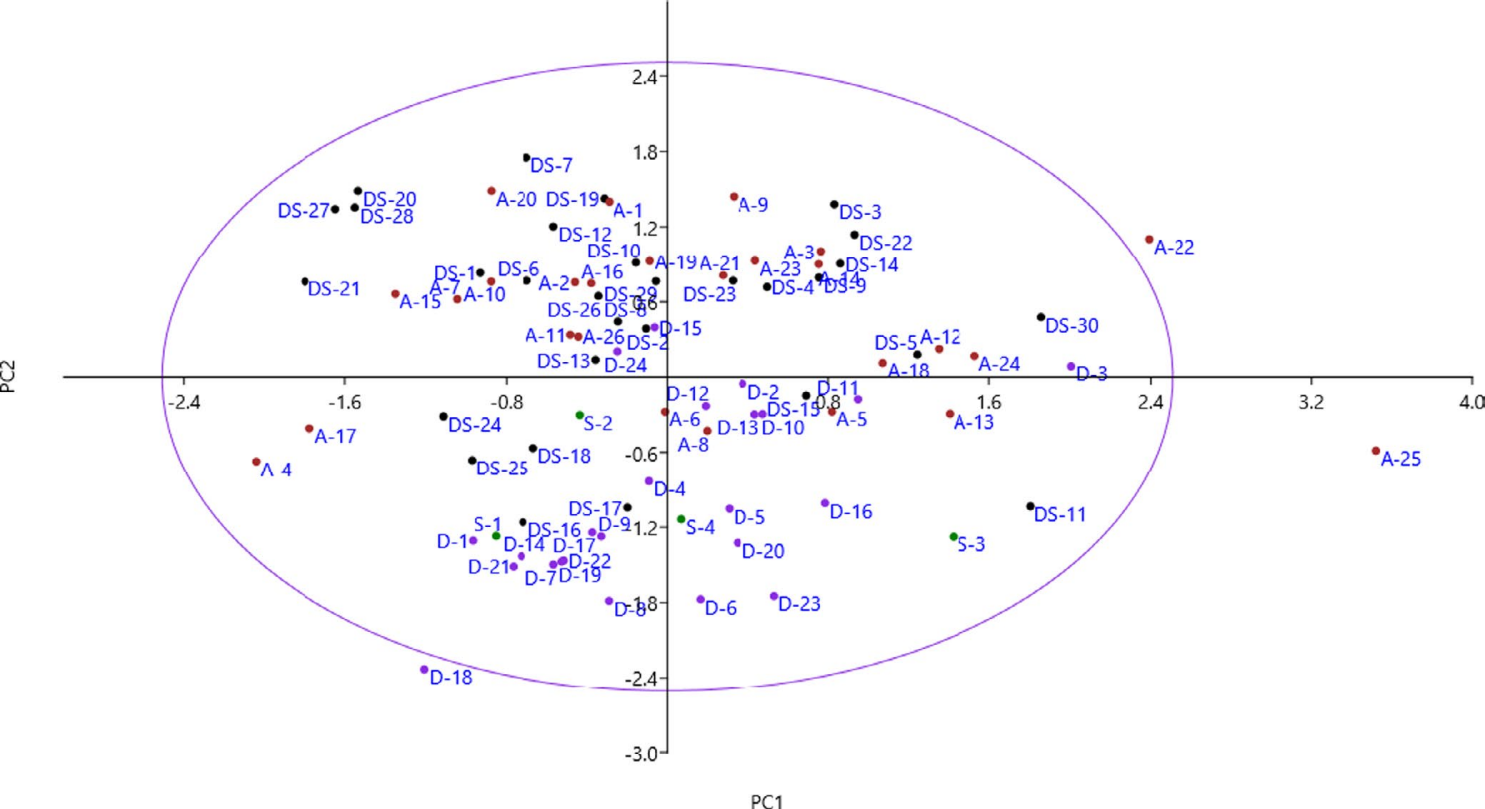
Scatterplot for the studied accessions of *E. angustifolia* based on PC1/PC2. The symbols represent the accessions of each area in the plot, including Astaneh (A), Dehmahdi (D), Dehsad (DS), and Senejan (S)

Bi‐plot analysis performed using the traits placed into PC1 and PC2 showed the relationships among the accessions, and the individuals within each of these PCs were more similar and grouped with each other. According to the duplicate analysis, the accessions were located on the four sides of the plot (Figure 2). In the bi‐plot, the accessions are represented in a two‐dimensional form and are shown based on the traits affecting PC1 and PC2.

**FIGURE 3:**
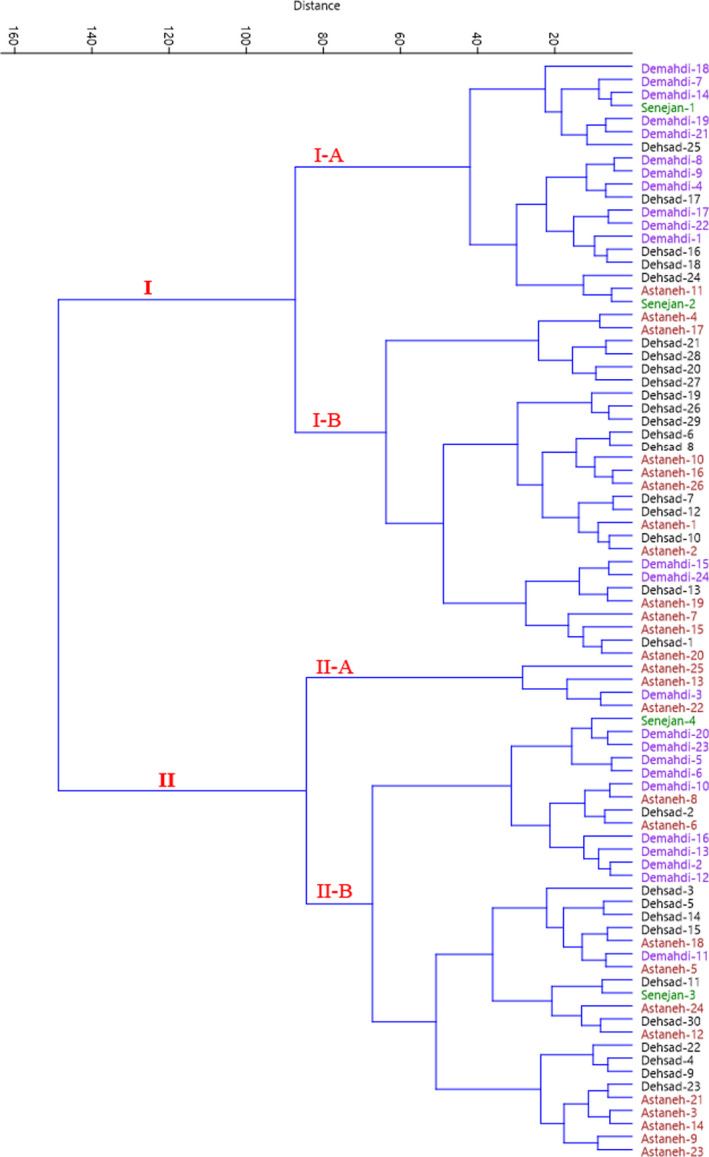
Ward cluster analysis of the studied accessions of *E. angustifolia* based on morphological traits using Euclidean distances

Cluster analysis is one of the methods in which several variables are used, and the purpose of this method is to classify individuals according to their characteristics. In the cluster analysis, the individuals within a class are highly similar to each other, with the highest nonuniformity and differences among clusters (Hair et al., 2009). In the cluster analysis based on Ward's method, the accessions were divided into two main groups according to morphological traits (Figure 3). The first group (I) included two subgroups; subgroup I‐A consisted of 19 accessions, and sub‐group I‐B contained 27 accessions. The rest accessions were classified as group II so that four accessions formed subgroup II‐A, and subgroup II‐B included 34 accessions.

**FIGURE 4:**
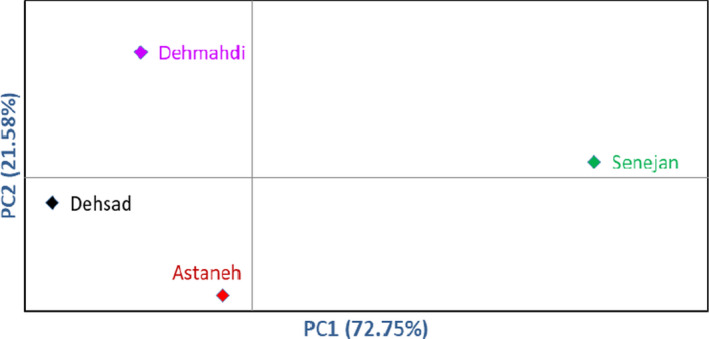
Bi‐plot for the studied areas of *E. angustifolia* based on morphological characters

In addition, the population analysis grouped the most similar areas within a group (Figure 4). The first cluster included the Senejan area, the second group consisted of Dehmahdi, and the third group included the Astaneh and Dehsad areas. There were great phenotypic variations among the accessions in terms of fruit‐related characters. The chance of genetic conservation, genotype selection, and improvement is significantly increased by enhancing knowledge on genotypic diversity (Wani et al., 2014). The variation observed in the same population can be due to the genetic and environmental effects (Karadeniz, 2002).

## 4 CONCLUSION

Significant differences were observed among the accessions of *E. angustifolia* based on the characters. Among the area studied, 14 accessions belonging to the Dehsad area, including Dehsad‐9, Dehsad‐10, Dehsad‐4, Dehsad‐27, Dehsad‐28, Dehsad‐29, Dehsad‐22, Dehsad‐3, Dehsad‐14, Dehsad‐7, Dehsad‐26, Dehsad‐23, Dehsad‐18, and Dehsad‐21, showed the highest value of fruit quality‐related characters, which are suitable for fresh consumption and health benefits. The results provided important information that is useful for selecting the preferred accessions for commercial cultivation and breeding programs.

## CONFLICT OF INTEREST

The authors declare no conflict of interest.

## AUTHOR CONTRIBUTIONS

**Leila Safdari:** Investigation (equal). **Ali Khadivi:** Formal analysis (equal); Investigation (equal); Validation (equal).

## DATA AVAILABILITY STATEMENT

The data that support the findings of this study are available from the corresponding author upon reasonable request.

5
